# Efficacy and safety of microbiota-targeted therapeutics in autoimmune and inflammatory rheumatic diseases: protocol for a systematic review and meta-analysis of randomised controlled trials

**DOI:** 10.1136/bmjopen-2025-101593

**Published:** 2025-12-14

**Authors:** Maja Skov Kragsnaes, Benoit Thomas P Gilbert, Bjørk Khaliqi Sofíudóttir, Christopher Michael Rooney, Sebrina Maj-Britt Hansen, Daniele Mauro, Benjamin H Mullish, Anne-Sophie Bergot, Kulveer Singh Mankia, Niti Goel, Gunnstein Bakland, Peter Holger Johnsen, Jesus Miguens Blanco, Simone Li, Emilie Dumas, Philip Rask Lage-Hansen, Carlijn Wagenaar, Ghaith Bakdash, Mat Robinson, Karsten Kristiansen, Julian R Marchesi, Georg Schett, Mario M Zaiss, Mine Orlu, Dirkjan van Schaadenburg, Jose U Scher, Dennis McGonagle, Dirk Elewaut, Maxime Breban, Peter Tugwell, Axel Finckh, Francesco Ciccia, Martin A Kriegel, Claire Daien, Torkell Ellingsen, Robin Christensen, EULAR Study Group MICMUC, Maja Skov Kragsnaes

**Affiliations:** 1 OUH Frontline Centre for Applied Microbiota and Mucosal Barrier Research (MICARE), Odense University Hospital, Odense, Denmark; 2 Research Unit of Rheumatology, Department of Clinical Research, University of Southern Denmark, Odense, Denmark; 3 Division of Rheumatology, HUG, Geneva, Switzerland; 4 Department of Rheumatology, Odense University Hospital, Odense, Denmark; 5 Department of Microbiology, Leeds Teaching Hospitals NHS Trust, Leeds, UK; 6 Leeds Institute of Medical Research, University of Leeds, Yorkshire, UK; 7 Open Patient data Explorative Network, Odense University Hospital, Odense, Denmark; 8 Department of Precision Medicine, University of Campania Luigi Vanvitelli, Naples, Italy; 9 Department of Metabolism, Digestion and Reproduction, Division of Digestive Diseases, Imperial College London, London, UK; 10 St Mary’s Hospital, Department of Gastroenterology and Hepatology, Imperial College Healthcare NHS Trust, London, UK; 11 Frazer Institute, The University of Queensland, Brisbane, Queensland, Australia; 12 Leeds Institute of Rheumatic and Musculoskeletal Medicine, NIHR Leeds Biomedical Research Centre, Leeds Teaching Hospitals NHS Trust, Leeds, UK; 13 Division of Rheumatology, Duke University School of Medicine, Durham, North Carolina, USA; 14 Department of Rheumatology, University Hospital of North Norway, Tromsø, Norway; 15 Department of Internal Medicine, University Hospital of North Norway, Harstad, Norway; 16 Microbiome Systems research group at the Biomedicine Discovery Institute, Monash University, Melbourne, Victoria, Australia; 17 Department of Rheumatology, University Hospital Ghent, Ghent, Belgium; 18 Center for Inflammation Research, Unit for Molecular Immunology and Inflammation, Flanders Institute for Biotechnology, Ghent, Belgium; 19 Department of Rheumatology, Esbjerg and Grindsted Hospital, Esbjerg, Denmark; 20 Amsterdam UMC, Amsterdam Rheumatology & Immunology Center, University of Amsterdam, Amsterdam, Netherlands; 21 Microbiotica, Chesterford Research Park, Little Chesterford, UK; 22 Laboratory of Integrative Biomedicine, Department of Biology, University of Copenhagen, Copenhagen, Denmark; 23 Friedrich-Alexander University Erlangen-Nuremberg, Erlangen, Germany; 24 Department of Internal Medicine 3, Universitätsklinikum Erlangen, Erlangen, Germany; 25 Department of Pharmaceutics, UCL School of Pharmacy, University College London, London, UK; 26 Department of Medicine, Division of Rheumatology, New York University Grossman School of Medicine, New York, New York, USA; 27 The Leeds Institute for Rheumatic and Musculoskeletal Medicine, University of Leeds, Leeds, UK; 28 Service de rhumatologie, Hôpital Ambroise Paré, Neuilly-sur-Seine, Île-de-France, France; 29 Université de Versailles St Quentin en Yvelines, Inserm, Infection et Inflammation, Université Paris-Saclay, Montigny le Btx, Île-de-France, France; 30 Department of Medicine, School of Epidemiology and Public Health, Bruyere Research Institute, Ottawa Hospital Research Institute, University of Ottawa, Ottawa, Ontario, Canada; 31 Institute of Musculoskeletal Medicine, Department of Translational Rheumatology and Immunology, University of Münster, Münster, Germany; 32 Department of Immunobiology, Yale University School of Medicine, New Haven, Connecticut, USA; 33 Department of Internal Medicine, Section of Rheumatology and Clinical Immunology, University Hospital Münster, Münster, Germany; 34 Department of Rheumatology, CHU de Montpellier, Montpellier, France; 35 the Parker Institute, Section for Biostatistics and Evidence-Based Research, Bispebjerg and Frederiksberg Hospital, Copenhagen, Denmark

**Keywords:** RHEUMATOLOGY, Microbiota, Antibiotics, Inflammation, Systematic Review, Meta-Analysis

## Abstract

**Abstract:**

**Introduction:**

An abnormal composition of gut bacteria along with alterations in microbial metabolites and reduced gut barrier integrity has been associated with the pathogenesis of chronic autoimmune and inflammatory rheumatic diseases (AIRDs). The aim of the systematic review, for which this protocol is presented, is to evaluate the clinical benefits and potential harms of therapies targeting the intestinal microbiota and/or gut barrier function in AIRDs to inform clinical practice and future research.

**Methods and analysis:**

This protocol used the reporting guidelines from the Preferred Reporting Items for Systematic Review and Meta-Analysis Protocol. We will search Embase (Ovid), Medline (Ovid) and the Cochrane Library (Central) for reports of randomised controlled trials of patients diagnosed with an AIRD. Eligible interventions are therapies targeting the intestinal microbiota and/or gut barrier function including probiotics, synbiotics, faecal microbiota transplantation, live biotherapeutic products and antibiotics with the intent to modify disease activity in AIRDs. The primary outcome of the evidence synthesis will be based on the primary endpoint of each trial. Secondary efficacy outcomes will be evaluated and selected from the existing core domain sets of the individual diseases and include the following domains: disease control, patient global assessment, physician global assessment, health-related quality of life, fatigue, pain and inflammation. Harms will include the total number of withdrawals, withdrawals due to adverse events, number of patients with serious adverse events, disease flares and deaths. A meta-analysis will be performed for each outcome domain separately. Depending on the type of outcome, the quantitative synthesis will encompass both ORs and standardised mean differences with corresponding 95% CIs.

**Ethics and dissemination:**

No ethics approval will be needed for this systematic review. We will follow the Preferred Reporting Items for Systematic Reviews and Meta-Analyses guidelines to disseminate the study results through a peer-reviewed publication.

**PROSPERO registration number:**

CRD42025644244.

**STRENGTHS AND LIMITATIONS OF THIS STUDY**

- This protocol follows Preferred Reporting Items for Systematic Reviews and Meta-analysis Protocols guidelines, ensuring a robust and standardised methodology.
- A predefined comprehensive search strategy across three databases combined with a search at ClinicalTrials.gov, as recommended by the Cochrane Musculoskeletal Group, and the WHO International Clinical Trial Registry Platform portal will be used to identify eligible trials.
- Due to an international author group, no language restrictions will be imposed on the selection of studies.
- Data on safety, primary trial endpoints and seven predefined efficacy outcomes reflecting the OMERACT core domains across chronic autoimmune and inflammatory rheumatic diseases will be systematically collected and presented separately.
- We will use the Grading of Recommendations Assessment, Development and Evaluation system to summarise the certainty of evidence, which will enhance the transparency and reliability of our conclusions.

## Introduction

 Within the last 25 years, the growing portfolio of biological agents and small molecule drugs that target specific cytokines and signalling pathways involved in the dysregulated immunological cascade of autoimmune and inflammatory rheumatic diseases (AIRDs) has greatly expanded the pharmacological toolbox.^1^ However, despite these breakthroughs in the management of AIRDs, current immunosuppressive therapies may still not be successful in achieving and/or maintaining long-standing disease control in a high proportion of treated patients.^2^,^4^ Suboptimal responses may reflect unknown environmental mediators of beneficial clinical responses despite maximal dose, tolerability issues and/or drug-related toxicity. Moreover, targeting several different cytokines or immune-related molecular pathways in combination may adversely affect host defence. Therefore, identification of new therapeutic targets that are central to the initiation and/or progression of AIRDs—but that do not require blocking of immunological pathways critical for host defence—is highly needed.

The human microbiota is one such promising therapeutic target. Although causality has yet to be established for most microbes associated with AIRDs, modulation of the intestinal microbiota is increasingly being recognised as a potential novel complementary therapy to immunomodulatory drugs.^5^ Many AIRDs are associated with gut microbial imbalances in both early and chronic disease stages,^6^,^10^ some of which have been mechanistically linked to disease pathogenesis.^11^,^13^ A systematic review of gut microbiota alterations in rheumatoid arthritis (RA), spondyloarthritis (SpA) and inflammatory bowel diseases (IBD) revealed reduced intestinal bacterial diversity and common features such as diminished Bacillota (formerly Firmicutes) abundance.^14^ Similarly, patients with systemic lupus erythematosus (SLE) exhibit overlapping features with IBD-associated microbiota. Additional changes include lower *Ruminococcaceae*, outgrowth of *Lactobacillus*, autoantigen cross-reactive pathobionts, as well as translocation of *Enterococcus gallinarum* and enrichment of *Ruminococcus gnavus*—the latter associated with lupus nephritis and SpA.^15^,^22^ In RA, disease-associated microbial expansions include *Segatella copri* (formerly *Prevotella copri*) in the gut and citrullination-promoting pathobionts in the oral cavity and intestine.^23^,^26^ Collectively, these findings underscore that perturbations across microbial taxa may influence autoimmune pathways and disease activity.

Research on gut microbiota modulation to promote human health is increasing.^27^ This systematic review focuses on gut microbiota-targeted therapeutic strategies and their potential application in AIRD disease management to facilitate evidence-based recommendations for clinical use and to guide future research priorities. Current interventions include personalised diets, prebiotics, probiotics, synbiotics, postbiotics, live biotherapeutic products, faecal microbiota transplantation (FMT), bacteriophages, microbiome mimetics and antibiotics,^28 29^ although not all have been explored in AIRDs. Among these, bacteriophages and microbiome mimetics represent emerging modalities under early investigation, while dietary interventions and prebiotics are considered beyond the scope of this review. The proposed therapeutic effects of microbiota-targeted therapies in AIRDs are thought to involve direct and indirect immunomodulatory mechanisms, including modulation of microbial composition and metabolites, restoration of the gut barrier integrity and/or alterations of host-drug interactions (pharmacomicrobiomics).^30 31^ Growing evidence of clinical benefits from probiotics^32^ and FMT^33^ in patients with IBD, alongside the overlapping gut microbiota alterations in AIRDs,^10 14 15^ has further encouraged clinical trials investigating the safety and efficacy of microbiota-targeted therapies in rheumatology.^34^,^36^

### Rationale

This review protocol is a collaborative initiative of the European Alliance of Associations of Rheumatology research study group on Microbiota and Mucosal Barrier Research in Rheumatic and Musculoskeletal Diseases (the MICMUC group). Established in 2022, the MICMUC group brings together patient partners, clinicians, translational scientists, statisticians and bioinformaticians in an international network dedicated to advancing research on microbial and mucosal barrier mechanisms in rheumatic diseases. Its main objectives include promoting multidisciplinary collaboration, identifying novel microbial biomarkers and therapeutic targets and evaluating the clinical potential of microbiota-targeted interventions and pharmacomicrobiomics.

To establish the need for a systematic review, we performed a pragmatic but systematic search of PubMed on 9 December 2024 to identify existing systematic reviews and meta-analyses. Of 1160 references identified, 246 were of potential interest based on title screening, excluding studies on unrelated diseases (eg, prosthetic joint, septic arthritis, osteoarthritis, gout) or interventions (eg, diet or prebiotics alone). After abstract screening, only 18 needed further scrutiny.^37^,^54^ Most of these reviews evaluated the efficacy of probiotics in RA.^37^,^4049 50 52^ The most recent and comprehensive review/meta-analyses on probiotics in inflammatory arthritis was based on reviews conducted in June 2020^40^ and May 2022,^52^ respectively. We also identified one meta-analysis on antibiotics for anti-neutrophil cytoplasmic antibody-associated vasculitis^44^ and a few systematic reviews on non-pharmacologic therapies in patients with SLE.^41^,^4346^ Only one review from 2022 evaluated efficacy and safety of FMT in AIRDs.^51^ Consequently, we decided that a comprehensive, updated investigation is warranted to guide patients and health professionals within this rapidly expanding research field.

### Objective

The main objective of this protocol is to outline the methods for conducting a systematic review and meta-analysis of randomised controlled trials (RCTs) evaluating the effectiveness and safety of therapies targeting the intestinal microbiota and/or gut barrier function in patients with AIRDs. Comparators will include inactive controls, established disease-modifying anti-rheumatic drugs (DMARDs) or other microbiota-targeted therapies. The secondary objective of the planned study is to assess the quality of trials fulfilling the eligibility criteria for study inclusion using the Cochrane Risk of Bias tool V.2.^55^

## Methods and analysis

### Methodological guidelines

The handbook guidance published by the Cochrane Collaboration (V.6, 2019)^56 57^ and the reporting guidelines for protocols of systematic reviews and meta-analyses (PRISMA-P) endorsed by the EQUATOR network have directed the planning and reporting of this protocol.^58^ In accordance with the guideline and prior to the conduct of the search and review process, the protocol was registered with the International Prospective Register of Systematic Reviews (registration date: 15 February 2025). The reporting of the results will follow the guidelines from EQUATOR on systematic reviews (PRISMA statement).^59^

### Criteria for selecting studies for this review

Studies will be selected according to the criteria defined below.

#### Study designs

Only RCTs will be considered for eligibility.

#### Participants

Eligible studies must include individuals diagnosed with one of the AIRDs listed in Box 1. To ensure consistency with rheumatology research practice while avoiding unnecessary exclusion of earlier trials, we will accept diagnoses based on recognised diagnostic criteria or established classification criteria available at the time the study was conducted. If no classification criteria were available for a given condition at the time, we will accept a clinical diagnosis, reflecting routine practice in early rheumatology intervention. No restrictions will be applied on age or sex/gender.

Box 1 **List of included autoimmune and inflammatory rheumatic diseases (AIRDs).**

AIRDs

Rheumatoid arthritis

Juvenile idiopathic arthritis

Axial spondyloarthritis

Psoriatic arthritis

Enteropathic arthritis

Reactive arthritis

Undifferentiated spondyloarthritis

Systemic lupus erythematosus

Antiphospholipid syndrome

Adult Still’s disease

Systemic sclerosis

Sjögren’s disease

Mixed connective tissue disease

Giant cell arteritis

Polymyalgia rheumatica

Takayasu arteritis

Polyarteritis nodosa

ANCA-associated vasculitis

Microscopic polyangiitis

Granulomatosis with polyangiitis

Eosinophilic granulomatosis with polyangiitis

Behçet’s disease

Anti-GBM disease

Cryoglobulinaemic syndrome

Polymyositis

Dermatomyositis

Clinical amyotrophic dermatomyositis

Inclusion body myositis

Antisynthetase syndrome

Eosinophilic myositis

Eosinophilic fasciitis

Relapsing polychondritis

Sarcoidosis

Periodic fever syndrome

Familial Mediterranean fever

TNF-receptor associated periodic syndrome

Cryopyrin associated periodic syndromes

#### Interventions

The therapies targeting the intestinal microbiota and/or gut barrier function being evaluated in this review encompass interventions administered to the gastrointestinal tract with the intention to treat AIRDs through modification(s) of the intestinal *milieu* (please see the definition of the selected therapies below). No restrictions are applied on dosage, frequency, duration of treatment or route of administration.

The microbiota-targeted interventions being evaluated are as follows:

1. Probiotics defined as live microorganisms that, when administered in adequate amounts, confer a health benefit on the host (eg, bacteria of the genera *Lactobacillus*, *Bifidobacterium*, *Enterococcus* or the species *Escherichia coli*).^60^
2. Synbiotics are defined as a combination of prebiotics and probiotics.
3. Postbiotics are defined as a preparation of inanimate microorganisms and/or their components that confers a health benefit on the host (ie, intact non-viable microbes or cell fragments, with or without metabolites such as heat-killed *Akkermansia muciniphila*).^61^
4. FMT products, encompassing minimally manipulated communities of intestinal micro-organisms from healthy individuals (donors).^62 63^
5. Live biotherapeutic products registered as medicinal products (pharmabiotics), defined as (1) containing live organisms, such as bacteria or bacterial spores; (2) applicable to the prevention, treatment or cure of a disease or condition of human beings and (3) not being a vaccine.^64^
6. Antibiotics are defined as a substance able to inhibit or kill microorganisms.

Studies of isolated diet interventions, prebiotics defined as substrates that are selectively utilised by host microorganisms conferring a health benefit (eg, fibres),^65^ and probiotics within functional food are excluded.

#### Comparator

All standard comparator types for the control groups such as inactive controls and established DMARDs will be included. Studies comparing two or more microbiota-targeted therapies without the use of a no-effect comparator will also be included.

### Outcomes

#### Primary outcome

For this systematic review, we prespecify the primary outcome as the trial-level measure that best reflects the efficacy of microbiota-targeted therapies for AIRDs, consistent with our review objectives and with Grading of Recommendations Assessment, Development and Evaluation (GRADE) guidance on defining outcomes relevant to the decision context.^66 67^ The primary outcome for the review is therefore *our* choice, not determined by trial authors. Because trials may report multiple candidate outcomes—and may label different endpoints as ‘primary’—we will use a predefined hierarchy to select the most appropriate measure from each study while limiting selective outcome reporting bias.^66^ The hierarchy is: the outcome reported as informing sample size calculation (trial’s most operational ‘primary’ measure); patient global assessment of improvement (if available); physician global assessment of improvement; the outcome judged by reviewers to best correspond to the review’s primary outcome domain.

This approach ensures that the review-defined primary outcome is consistently extracted across heterogeneous trials while minimising indirectness.

#### Secondary outcomes

We also pre-specify the secondary outcomes for the review, reflecting domains most relevant to the effects and safety of microbiota-targeted therapies in AIRDs. Because no cross-disease core outcome measurement set exists for AIRDs,^68^ and no harmonised core safety set has been finalised for rheumatology trials,^69^ we draw on the Outcome Measures in Rheumatology (OMERACT)-endorsed core domain framework.^70^ Secondary outcomes will therefore include relevant domains such as disease activity, patient global assessment, physician global assessment, quality of life, fatigue, pain, inflammation and adverse events. Trials are eligible for the review regardless of which outcomes they report. However, a study will contribute to a given meta-analysis only if it provides complete extractable data (numerical results or sufficiently detailed figures). When outcomes are reported as composite measures, both the composite and its components will be extracted as presented. For each domain, we will extract only one pre-specified measurement instrument to avoid unit-of-analysis issues.^71^

Secondary efficacy outcomes: hierarchy and definitions

1. Disease control defined as clinical remission, or alternatively (very) low disease activity

RA: for example, DAS28(CRP) <2.6

SLE: for example, Definition Of Remission In SLE, clinical Systemic Lupus Erythematosus Disease Activity Index-2K (SLEDAI-2K)=0, lupus low disease activity state, SLEDAI-2K≤4

All AIRDs: for example, a proxy for disease control such as no need to escalate guideline therapy

1. Patient global assessment of disease activity^72^

For example, patient global assessment of disease activity on a visual analogue scale (VAS)

1. Physician global assessment

For example, physician global assessment of disease activity on VAS

1. Health-related quality of life

For example, listed in the order of preference

- The Medical Outcomes Study Short Form 36 (SF-36).
  - Physical component (SF-36 PCS).
  - Mental component (SF-36 MCS).
- EuroQol 5-domain (EQ-5D).

1. Fatigue

For example,

- Multidimensional Assessment of Fatigue scale.
- Multi-dimensional Fatigue Index.
- The Fatigue Severity Scale (FSS).
- The Functional Assessment of Chronic Illness Therapy–Fatigue (FACIT-F) scale.
- The Brief Fatigue Inventory.
- The vitality scale of the SF-36.
- The visual analogue scale for vitality.

1. Pain^7^

- For example, pain on VAS.

1. Systemic inflammation

For example,

- C reactive protein.
- Erythrocyte sedimentation rate.
- Blood cytokine levels.
- Numbers or proportions of immune cells.

Safety outcomes

I. Total number of withdrawals.
II. Withdrawals due to adverse events.
III. Number of patients with serious adverse events (SAEs).
IV. Number of deaths.

### Timing of outcome evaluation

The preferred time point of measurements is the last day of the intervention or as late as possible when the participants still receive the intervention. In trials where the intervention is only performed once or a few times at the beginning of the trial (eg, FMT), the original trial design will be used. Trusting that most trials have been pre-specified and appropriately registered, the trial’s time point for the primary outcome evaluation will be used for the evaluation of effectiveness outcomes in the present meta-analysis. Safety measures will be evaluated as late as possible (ie, while still respecting the original design).

### Setting

There will be no restrictions by type of setting.

### Language

No language restrictions will be imposed, although only studies in languages other than English that can be translated adequately by co-authors or by using Google Translate or other free accessible resources will be included, due to resource limits. A list of possibly relevant titles in languages that we did not manage to translate will be provided as an appendix.

### Publication types

Both full-text manuscripts and abstracts will be included, including conference abstracts and abstracts for which the full text could not be obtained despite contacting the authors.

### Information sources

The systematic search will be performed using three bibliographic databases: Embase (Ovid), Medline (Ovid) and Cochrane Library (Central). In addition, a search will be conducted in ClinicalTrials.gov and the WHO International Clinical Trial Registry Platform portal (ICTRP) as recommended by the Cochrane Musculoskeletal Group^68^ from inception of the data source to the study search date. After the inclusion of eligible studies, a backward and forward citation will be conducted in Scopus for twenty randomly selected reports. If no additional relevant records are identified, the search will be considered comprehensive. If new studies are found, full citation tracking will be conducted for all included reports to capture any remaining relevant records.

### Search strategy

The search strategy was developed by BS and MSK in collaboration with an information specialist. An initial search of PubMed was undertaken, followed by an analysis of the text words contained in the title and abstract, and of the index terms used to describe articles. The search strategy was developed using a 15-step methodology recommended by Bramer *et al*^74^ The search strategy was first tested in Embase as recommended by Bramer *et al*, and adjusted according to new keywords and subject headings across Medline and Cochrane Central. The search strategy will be reported according to the PRISMA-S guideline for reporting searches (https://www.prisma-statement.org/prisma-search). The preliminary full search strategy for Embase is presented in the online supplemental file 1. This strategy will serve as the template for searches in the other databases, with modifications made to reflect database-specific indexing terms (eg, MeSH in Medline) and search syntax.

### Study records

#### Study selection process

**Figure 1:**
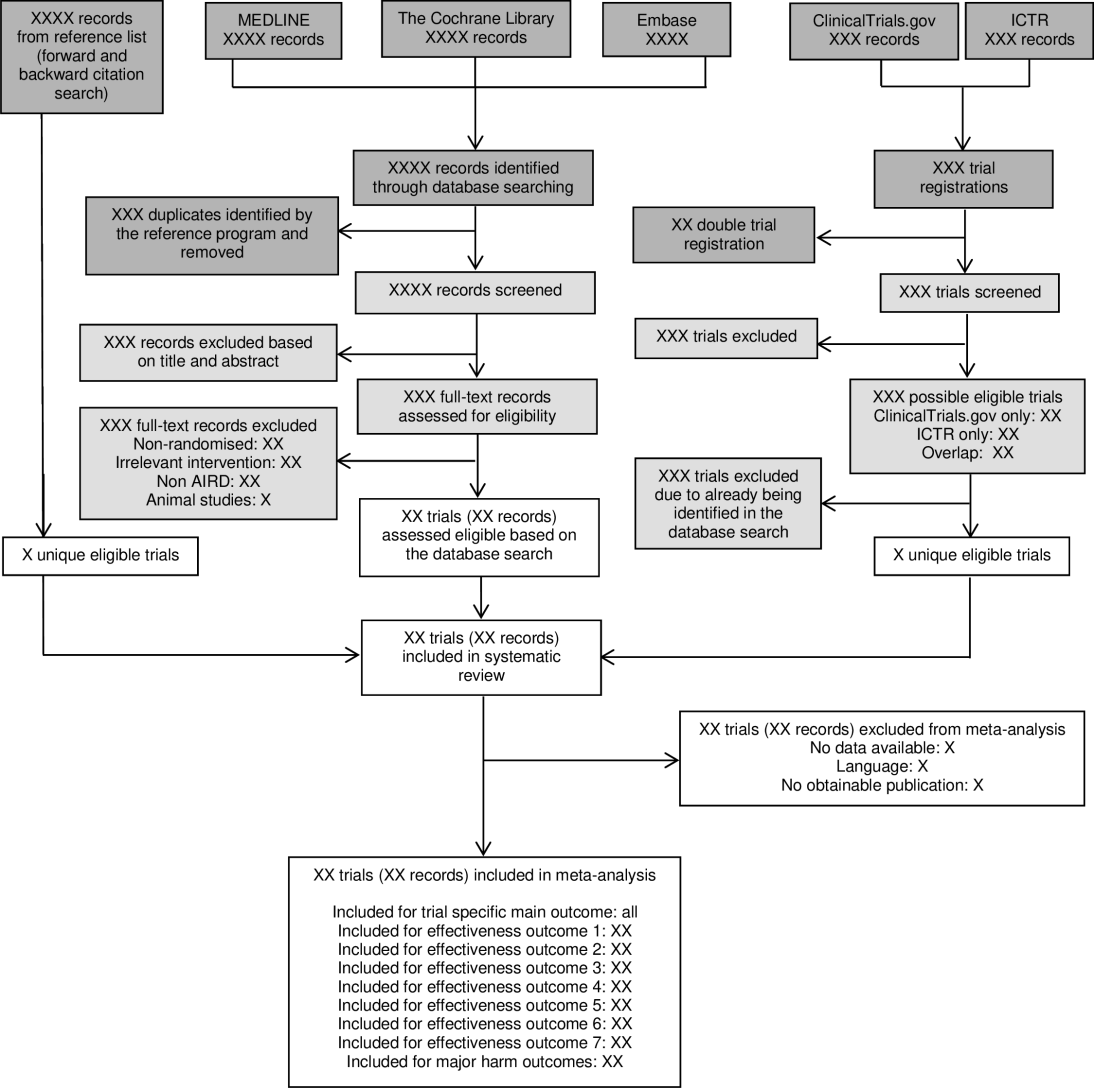
Flow diagram showing the selection of trials. Boxes are shaded dark grey (study identification), light grey (screening) and white (inclusion of final reports for the systematic review and meta-analysis) to illustrate the three stages outlined in the PRISMA 2020 flow diagram. Studies available only as abstracts will be considered for inclusion; however, they will be included in the meta-analysis only if sufficient data are available for analysis. AIRD, autoimmune and inflammatory rheumatic disease. ICTRP, WHO International Clinical Trial Registry Platform portal; PRISMA, Preferred Reporting Items for Systematic Reviews and Meta-Analyses.

The search results will be managed in Covidence, which will be used for deduplication and reference screening. Records retrieved through the search strategy will be imported into Covidence by an information specialist and two reviewers. Two reviewers will independently screen titles and abstracts to identify potentially eligible studies or where eligibility is uncertain. Full-text articles will then be reviewed in detail to assess inclusion (figure 1). Reasons for exclusion will be recorded. The two reviewers will meet in person to compare selected reports, and any disagreements will be resolved through discussion or, if necessary, by consulting a third reviewer. Reviewers will not be blinded to journal titles, study authors or institutions.

#### Data collection process

Full-text articles deemed eligible after screening will undergo in-depth assessment by two independent reviewer groups (each comprising two reviewers). Data from each included trial will be entered into a customised REDCap database,^75^ which will be designed so that data extracted from each trial will be entered into two similar, but separated records. Each group will have access only to its own record until data extraction is complete. The two review groups will independently extract data and assess risk of bias. Reviewers’ disagreements will be resolved through discussion within and between groups, both in person and during online meetings. If consensus cannot be reached, a fifth, blinded reviewer will adjudicate. If necessary, authors of included studies will be contacted to obtain missing or unpublished data.

Before commencing formal data extraction, all reviewers will undergo structured training using a small set of randomly selected trials not included in the final analysis. This pilot phase will ensure consistency in data extraction, calibration of the customised REDCap form and harmonisation of interpretation across reviewer groups. Discrepancies identified during the pilot will be discussed collectively, and the data extraction form will be refined as necessary before full data extraction begins.

#### Data items

We will extract two main types of information from each included trial:

1. Study characteristics and methods: title, year of publication, journal, author names, trial design (eg, parallel, cross-over, adaptive), group allocation, blinding methods and processes, duration of follow-up, time and reasons for trial termination (if applicable) and types of statistical analyses performed. Participant characteristics will include age, sex, diagnosis, disease duration, disease status (eg, active disease, remission), concurrent anti-inflammatory medication (including dosage, frequency and timing relative to the experimental intervention) and country of trial conduct. Financial support sources will also be recorded.
2. Intervention and outcome data: details of the experimental and control interventions, including generic and trade names, route of administration, dosage, frequency, duration of treatment, single versus mixed donor (for FMT) and control type. Outcome data will include the definition of the primary endpoint as reported in the trial, secondary outcomes and all reported outcome domains. Where necessary, summary statistics will be approximated from figures. For cross-over trials, only first-period data will be extracted to avoid carry-over effects. Response rates will be calculated based on the total number of randomised participants (intention-to-treat population), and we will prioritise intention-to-treat results whenever available.

### Risk of bias assessment in the individual trials

Eligible trials will be assessed independently pairwise by four reviewers for methodological issues using the Cochrane Risk of Bias tool V.2.^55^ This risk of bias tool covers six domains of bias: selection bias, performance bias, detection bias, attrition bias, reporting bias and other bias. For each item in the tool, the assessment of risk of bias is in two parts (risk judgement and trial description that supports this judgement). The support for judgement provides a concise free text description or summary of the relevant trial characteristic on which judgments of risk of bias are based and aims to ensure transparency in how judgments are reached. Any disagreements that arise between the reviewers will be resolved by consensus or by consulting a fifth reviewer. Response options (ie, judgments about risk) are low risk, high risk and unclear risk. Hence, studies will not be excluded on the results of critical appraisal; however, study quality will be considered when analysing and interpreting results.

### Data synthesis

#### Primary and secondary endpoint analyses

Meta-analyses will be conducted separately for each primary and secondary outcome domain. For trials with multiple intervention arms, the number of patients in the shared control group will be divided by the number of comparisons to avoid double-counting and to produce correct estimates with appropriately increased standard errors.

Binary outcomes^66^: Most primary endpoints are anticipated to be binary. ORs will be computed such that a result greater than one indicates greater effectiveness of microbiota-targeted therapeutics compared with placebo/sham. In trials utilising continuous outcomes instead of discrete ones, if the means and SD of the placebo and treatment groups on these trial outcomes are available, they will be transformed into the corresponding OR using a method outlined by Chinn.^76^ Whenever feasible, all participants who drop out will be considered as non-responders, adhering to the intention-to-treat principle. ORs are also calculated for withdrawals, withdrawals due to adverse events, number of SAEs and deaths.

Continuous outcomes^77^: For continuous outcome domains, standardised mean differences (SMDs) will be used to combine results measured with different instruments. Mean differences (MD) at follow-up will be used when the change from baseline is not available. SMDs are calculated by dividing the difference in mean values by the pooled SD for the given outcome; a correction will be applied by default by calculating Hedges’s g^78^ and the variance (SE^2^) will be calculated based on the SMD and number of patients in each group. This calculation involves dividing the difference between the intervention and comparator mean changes in each trial (ie, the MD by the estimated within-group SD for that trial). Accordingly, an SMD <zero will indicate a beneficial effect of the experimental intervention (eg, a reduction in the patient’s global assessment) compared with control comparator. The SMD will be converted to ORs by the conversion proposed by Hasselblad and Hedges: ln(OR)= SMD $\frac{\pi }{\surd 3}$

#### Evidence synthesis methods

All meta-analyses will be performed using R Foundation for Statistical Computing or Stata, with effect estimates pooled using random-effects models based on restricted maximum likelihood (REML). Fixed-effect models will be used in sensitivity analyses to test the robustness of results. Statistical heterogeneity will be investigated using standard Forest plots for each domain, and the heterogeneity will be evaluated by inconsistency index (I^2^ statistic).

#### Subgroup, meta-regression and sensitivity analyses

To investigate possible sources of trial heterogeneity, additional analyses of the main outcome will be performed. We will stratify the available trials according to trial characteristics: (1) type of diagnosis, (2) type of intervention and (3) type of concurrent anti-inflammatory treatment, respectively, using univariate REML-based meta-regression. We will also explore the statistical effect of trial design according to the following subgroups: (I) Trials at risk of bias (on an item-by-item basis); (II) trials with shorter vs longer follow-up and (III) Comparator (active vs placebo).

#### Dealing with missing data

When there are missing data, we will attempt to contact the authors of the study to obtain the relevant missing data. Important numerical data will be carefully evaluated. If missing data cannot be obtained, an imputation method will be used. Anticipating ROB-ME (risk of bias due to missing evidence), the structured approach for assessing the risk of bias that arises when entire studies, or particular results within studies, are missing from a meta-analysis because of the p-value, magnitude or direction of the study results.^67^ To visualise the potential for bias in the meta-analysis result, we will generate Forest plots displaying trials with results, along with information on trials that did not report specific outcome domains.

### Rating certainty of the evidence

The GRADE system will be used^79^ to summarise the certainty of evidence on an outcome-by-outcome basis as high, moderate, low or very low.^80^ Quality of evidence will not be downgraded for risk of bias if subgroup analysis indicates no association of treatment effects with risk of bias. Additionally, when there are a minimum of 10 studies for meta-analysis, we will attempt to evaluate the risk of publication bias through visual assessment of funnel plot asymmetry.

### Presentation of results

Depending on the type of outcome, the quantitative synthesis will encompass both ORs and SMD with corresponding 95% CIs for each outcome domain. Forest plots will be used for graphical representation. Exact p values will be reported, and statistical significance will be defined as p<0.05. Sensitivity and subgroup analyses will be reported alongside the main analyses to allow full interpretation of the robustness of findings.

### Protocol deviations

Any modifications to this protocol during the study’s execution will be documented in the final manuscript and on PROSPERO.

### Study timeline

The anticipated timeline for conducting this systematic review and meta-analysis is outlined below:

- Development and finalisation of search strategy: by December 2025.
- Title and abstract screening: by February 2026.
- Full-text review and data extraction: by June 2026.
- Statistical analysis and synthesis of results: by October 2026.
- Manuscript preparation and dissemination of findings: by December 2026.

## Patient and public involvement

A patient partner (NG) was involved in the design, conduct, reporting and dissemination plans of this research.

## Ethics and dissemination

No ethical approval will be needed because we will be using data from previously published studies. The results will be disseminated through presentations at scientific conferences and publication in a peer-reviewed journal. When interpreting the knowledge arising from the systematic review, we will evaluate possible limitations at both the study level, such as risk of bias relating to randomisation and allocation to treatment groups or missing data, and the review level, such as selection bias due to different inclusion criteria and potential heterogeneity resulting from differences in disease, intervention, treatment comparators, outcome and follow-up points.

## Discussion

By conducting this systematic review and meta-analysis, we aim to synthesise the existing evidence from RCTs on the clinical efficacy and safety of microbiota-targeted therapies in AIRDs. This includes interventions capable of modulating the human microbiota, such as probiotics, synbiotics, FMT, biotherapeutic products and antibiotics with immunomodulatory properties.^81^ While antibiotics are well-defined drug classes, microbial-based therapies lack standardisation due to regulatory and manufacturing variations,^6482^,^84^ which may limit direct comparisons across trials. By systematically extracting and analysing trial data—including intervention characteristics, dosage, administration and outcomes—we will provide quantitative estimates of effect for both primary and secondary endpoints. This approach will enable a rigorous evaluation of the evidence base, clarify knowledge gaps and inform future clinical guidance on microbiota-targeted therapies in patients with AIRD.

Despite great variability in genetic dispositions, disease type as well as disease stage (preclinical phase, early manifest disease and chronic disease stages), which have been associated with different alterations in microbial composition and function in AIRDs, a common denominator across these distinct disease entities seems to be a reduction in bacterial diversity and a low abundance of microbes producing short-chain fatty acids (SCFAs). SCFAs are microbiota-derived metabolites that are involved in many physiological pathways, including the strengthening of the gut barrier integrity.^85^ Notably, several studies have documented increased intestinal permeability in both RA,^86^ SpA,^87^ psoriatic arthritis^88^ and SLE,^1889^,^91^ which seems to be related to disease activity. These shared microbiota-related features are the main reason for including all AIRDs in one systematic review. Still, we acknowledge that randomised trials encompassing patients with relatively rare AIRDs, such as relapsing polychondritis and cryopyrinopathies, may be limited.

Although mode(s) of action of microbiota-targeted interventions in AIRD are still not clear, one of the primary aims of these interventions is to reduce systemic inflammation by boosting healthy microbial diversity, increasing the number of microbes producing SCFA,^85^ and/or eliminating proposed disease-causing, pathogenic ones. Other effects of microbiota-targeted therapies may be mediated through pharmacomicrobiomic interactions, where changes in the intestinal microbiota directly modulate drug disposition, action and/or toxicity.^92^ Interestingly, gut bacterial compositions in patients prior to treatment instigation have been shown to relate to clinical response to conventional therapies such as methotrexate, sulfasalazine and biological drugs.^93^,^96^ Therefore, the current review will also present data on any concurrent immunomodulatory drugs used in combination with the experimental microbiota-targeted therapeutic.

While exposure to most antibiotics reduces the overall bacterial diversity both in the short and long term^97^—and its use has been associated with the development of arthritis^98 99^ (but also resolution in rare cases)^100^—some antibiotics have been attributed immunomodulatory effects,^101 102^ which have motivated trials evaluating their potential disease-modifying effects in AIRDs. In addition, some microbiota-targeted strategies encompass pre-conditioning with antibiotics aiming at resetting the recipient’s microbiota before the application of probiotics and/or FMT. Even though this approach may adversely affect microbiota at mucosal sites, without the possibility for subsequent restoration of healthy microbiota (eg, pulmonary microbiota), trials evaluating such combinations of microbiota-targeted therapies will also be included.

People living with AIRDs often experience that the disease impacts their lives in multiple ways. Consequently, the core outcome set in rheumatology represents the minimum set of domains and measurement instruments that should be reported in every trial of a specific condition.^103^ Therefore, we will not only extract data on the predefined primary endpoint of each trial, but also collect and present data on available outcomes from preselected OMERACT core domain sets addressing both disease control, the physician and patient global assessment of disease, quality of life, fatigue, pain and inflammation. We acknowledge that some of the trials that will be included in this review have been conducted before the widespread adoption of these outcome sets, which may limit the amount of accessible data that can be retrieved for one or more of these selected domains. However, all results will be interpreted carefully, taking this as well as other limitations into account.

In conclusion, this systematic review and meta-analysis aims to provide a robust and comprehensive synthesis of current evidence regarding the efficacy and safety of microbiota-targeted therapies in AIRDs. The results will support the development of evidence-based clinical recommendations and inform future research directions in this emerging area.

## Supplementary material

10.1136/bmjopen-2025-101593 online supplemental file 1

## Data Availability

No data are available.
