# Supplementary material for: Efficacy and safety of microbiota-targeted therapeutics in autoimmune and inflammatory rheumatic diseases: protocol for a systematic review and meta-analysis of randomised controlled trials
Source: BMJ Open. 2025 Dec 14;15(12):e101593. doi: 10.1136/bmjopen-2025-101593 (PMC12706220; doi:10.1136/bmjopen-2025-101593)
Supplement: online supplemental file 1 [file bmjopen-15-12-s001.pdf]

Supplemental S1 Draft search strategy for Embase

arthritis/
antisynthetase syndrome/
behcet disease/
chronic arthritis/
reiter syndrome/
exp monarthritis/
exp polyarthritis/
exp rheumatoid arthritis/
spondylitis/
exp spondylarthritis/
exp systemic lupus erythematosus/
exp antiphospholipid syndrome/
exp systemic sclerosis/
Sjoegren syndrome/
mixed connective tissue disease/
relapsing polychondritis/
temporal arteritis/
rheumatic polymyalgia/
aortic arch syndrome/
large vessel vasculitis/
exp ANCA associated vasculitis/
microscopic polyangiitis/
cryoglobulinemia/
exp polymyositis/
exp dermatomyositis/
exp inclusion body myositis/
familial Mediterranean fever/
CINCA syndrome/
exp tumor necrosis factor receptor associated periodic syndrome/
polyarteritis nodosa/
arthritis.ti,ab.
antisynthetase syndrome.ti,ab.
enthesitis.ti,ab.
rheumatic* disease.ti,ab.
rheumatism.ti,ab.
spondyloarthropathies.ti,ab.
spondylitis.ti,ab.
spondylarthr*.ti,ab.
spondyloarthr*.ti,ab.
ankylosing spondylitis.ti,ab.
uspa.ti,ab.
reiter's.ti,ab.
systemic lupus erythematosus.ti,ab.
antiphospholipid syndrome.ti,ab.
still* disease.ti,ab.
systemic sclero*.ti,ab.

sjogren* syndrome.ti,ab.
relapsing polychondritis.ti,ab.
giant cell arteritis.ti,ab.
polymyalgia rheumatic*.ti,ab.
takayasu arteritis.ti,ab.
aortic arch syndrome.ti,ab.
large vessel vasculitis.ti,ab.
anca associated vasculitis.ti,ab.
anti Neutrophil cytoplasmic antibody vasculitis.ti,ab.
microscopic polyangiitis.ti,ab.
granulomatosis with polyangiitis.ti,ab.
wegener granulomatosis.ti,ab.
eosinophilic granulomatosis with polyangiitis.ti,ab.
polyarteritis nodosa.ti,ab.
behcet* disease.ti,ab.
behcet* syndrome.ti,ab.
anti GBM antibod*.ti,ab.
cryoglobulinemia*.ti,ab.
polymyositis.ti,ab.
dermatomyositis.ti,ab.
antisynthetase syndrome.ti,ab.
eosinophilic myositis.ti,ab.
periodic fever syndrome*.ti,ab.
familial mediterranean fever.ti,ab.
tnf receptor-associated syndrome.ti,ab.
tumor necrosis factor receptor-associated syndrome.ti,ab.
cryopyrin associated-syndrome*.ti,ab.
cinca syndrome.ti,ab.
AIMD.ti,ab.
polyarteritis nodosa.ti,ab.
(systemic adj1 sclero*).ti,ab.
(relapsing adj1 polychondritis).ti,ab.
(polymyalgia adj1 rheumatic*).ti,ab.
(anti glomerular basement membrane adj2 antibod*).ti,ab.
(inclusion body adj1 myositis).ti,ab.
(tumor necrosis factor receptor associated adj2 syndrome).ti,ab.
(cryopyrin associated adj2 syndrome*).ti,ab.
(sjogren* adj2 syndrome).ti,ab.
(rheumatic* adj2 disease).ti,ab.
(polymyalgia adj1 rheumatic*).ti,ab.
(behcet* adj2 disease).ti,ab.
(behcet* adj2 syndrome).ti,ab.
(antisynthetase adj2 syndrome).ti,ab.
cryopyrin-associated periodic syndrome*.ti,ab.
arthritis/
antisynthetase syndrome/
behcet disease/
chronic arthritis/
reiter syndrome/

exp monarthritis/
exp polyarthritis/
exp rheumatoid arthritis/
spondylitis/
exp spondylarthritis/
exp systemic lupus erythematosus/
exp antiphospholipid syndrome/
exp systemic sclerosis/
Sjoegren syndrome/
mixed connective tissue disease/
relapsing polychondritis/
temporal arteritis/
rheumatic polymyalgia/
aortic arch syndrome/
large vessel vasculitis/
exp ANCA associated vasculitis/
microscopic polyangiitis/
cryoglobulinemia/
exp polymyositis/
exp dermatomyositis/
exp inclusion body myositis/
familial Mediterranean fever/
CINCA syndrome/
exp tumor necrosis factor receptor associated periodic syndrome/
polyarteritis nodosa/
arthritis.ti,ab.
antisynthetase syndrome.ti,ab.
enthesitis.ti,ab.
rheumatic* disease.ti,ab.
rheumatism.ti,ab.
spondyloarthropathies.ti,ab.
spondylitis.ti,ab.
spondylarthr*.ti,ab.
spondyloarthr*.ti,ab.
ankylosing spondylitis.ti,ab.
uspa.ti,ab.
reiter's.ti,ab.
systemic lupus erythematosus.ti,ab.
antiphospholipid syndrome.ti,ab.
still* disease.ti,ab.
systemic sclero*.ti,ab.
sjogren* syndrome.ti,ab.
sjoegren* syndrom.ti,ab.
mixed connective tissue disease.ti,ab.
relapsing polychondritis.ti,ab.
giant cell arteritis.ti,ab.
polymyalgia rheumatic*.ti,ab.
takayasu arteritis.ti,ab.
aortic arch syndrome.ti,ab.

large vessel vasculitis.ti,ab.
anca associated vasculitis.ti,ab.
anti Neutrophil cytoplasmic antibody vasculitis.ti,ab.
microscopic polyangiitis.ti,ab.
granulomatosis with polyangiitis.ti,ab.
wegener granulomatosis.ti,ab.
eosinophilic granulomatosis with polyangiitis.ti,ab.
polyarteritis nodosa.ti,ab.
behcet* disease.ti,ab.
behcet* syndrome.ti,ab.
anti GBM antibod*.ti,ab.
anti glomerular basement membrane antibod*.ti,ab.
cryoglobulinemia*.ti,ab.
polymyositis.ti,ab.
dermatomyositis.ti,ab.
inclusion body myositis.ti,ab.
antisynthetase syndrome.ti,ab.
eosinophilic myositis.ti,ab.
periodic fever syndrome*.ti,ab.
familial mediterranean fever.ti,ab.
tnf receptor associated syndrome.ti,ab.
tumor necrosis factor receptor associated syndrome.ti,ab.
cryopyrin-associated syndrome*.ti,ab.
cinca syndrome.ti,ab.
AIMD.ti,ab.
polyarteritis nodosa.ti,ab.
(systemic adj1 sclero*).ti,ab.
(relapsing adj1 polychondritis).ti,ab.
(polymyalgia adj1 rheumatic*).ti,ab.
(anti glomerular basement membrane adj2 antibod*).ti,ab.
156 or 174
(inclusion body adj1 myositis).ti,ab.
160 or 176
(tumor necrosis factor receptor associated adj2 syndrome).ti,ab.
(cryopyrin associated adj2 syndrome*).ti,ab.
(sjogren* adj1 syndrome).ti,ab.
(sjogren* adj2 syndrome).ti,ab.
(rheumatic* adj1 disease).ti,ab.
(rheumatic* adj2 disease).ti,ab.
(polymyalgia adj1 rheumatic*).ti,ab.
(behcet* adj1 disease).ti,ab.
(behcet* adj2 disease).ti,ab.
(behcet* adj1 syndrome).ti,ab.
(behcet* adj2 syndrome).ti,ab.
(antisynthetase adj2 syndrome).ti,ab.
cryopyrin-associated periodic syndrome*.ti,ab.
1 or 2 or 3 or 4 or 5 or 6 or 7 or 8 or 9 or 10 or 11 or 12 or 13 or 14 or 15 or 16 or 17 or 18 or 19 or 20 or 21 or 22 or 23 or 24 or 25 or 26 or 27 or 28 or 29 or 30 or 31 or 32 or 33 or 34 or 35 or 36 or 37 or 38 or 39 or 40 or 41 or 42 or 43 or 44 or 45 or 46 or 47 or 48 or 49 or 50 or 51 or 52 or 53 or 54 or 55 or 56 or 57 or 58 or 59 or 60 or 61 or 62 or 63 or 64 or 65 or 66 or 67 or 68 or 69 or 70 or 71 or 72 or 73

or 74 or 75 or 76 or 77 or 78 or 79 or 80 or 81 or 82 or 83 or 84 or 85 or 86 or 87 or 88 or 89 or 90 or 91 or 92 or 93 or 94 or 95 or 96 or 97 or 98 or 99 or 100 or 101 or 102 or 103 or 104 or 105 or 106 or 107 or 108 or 109 or 110 or 111 or 112 or 113 or 114 or 115 or 116 or 117 or 118 or 119 or 120 or 121 or 122 or 123 or 124 or 125 or 126 or 127 or 128 or 129 or 130 or 131 or 132 or 133 or 134 or 135 or 136 or 137 or 138 or 139 or 140 or 141 or 142 or 143 or 144 or 145 or 146 or 147 or 148 or 149 or 150 or 151 or 152 or 153 or 154 or 155 or 156 or 157 or 158 or 159 or 160 or 161 or 162 or 163 or 164 or 165 or 166 or 167 or 168 or 169 or 170 or 171 or 172 or 173 or 174 or 175 or 176 or 177 or 178 or 179 or 180 or 181 or 182 or 183 or 184 or 185 or 186 or 187 or 188 or 189 or 190

fecal microbiota transplantation/

bacteriotherapy/

exp probiotic agent/

fecal microbiota transplant*.ti,ab.

faecal microbiota transplant*'.ti,ab.

fecal microbiome transplant*.ti,ab.

faecal microbiome transplant*.ti,ab.

stool transplant*.ti,ab.

fmt.ti,ab.

fecal transfusion*.ti,ab.

fecal bacteriotherap*.ti,ab.

bacteriotherap*.ti,ab.

colonic restoration.ti,ab.

flora reconstitution.ti,ab.

achim.ti.ab,

RBX2660.ti,ab.

((Fecal or Faecal or microbiota or microflora or feces or faeces or stool) adj3 (transplant* or transfus* or implant* or instillation or donor*or enema or reconstitution orinfusion* or therap* or transfer* or treat*)).ti,ab.

exp antiinfective agent/

exp *antiinfective agent/

208 or 209

antibiotic*.ti,ab.

anti-biotic*.ti,ab.

antibacterial*.ti,ab.

anti-bacterial*.ti,ab.

antimicrobial*.ti,ab.

anti-microbial*.ti,ab.

antiseptic*.ti,ab.

anti-septic*.ti,ab.

bactericid*.ti,ab.

bacteriocid*.ti,ab.

bacteriostatic.ti,ab.

ciprofloxacin.ti,ab.

metronidazole.ti,ab.

levamisole.ti,ab.

ornidazole.ti,ab.

fusidin.ti,ab.

rifaximin.ti,ab.

vancomycin.ti,ab.

fusidic acid.ti,ab.

nitazoxanide.ti,ab.

teicoplanin.ti,ab.

rifampicin.ti,ab.

bacitracin.ti,ab.
fidaxomicin.ti,ab.
amoxicillin.ti,ab.
azithromycin.ti,ab.
cephalosporin*.ti,ab.
cephalexin.ti,ab.
clarithromycin.ti,ab.
clindamycin.ti,ab.
doxycycline.ti,ab.
erythromycin.ti,ab.
flouroquinolone*.ti,ab.
levofloxacin.ti,ab.
macrolide*.ti,ab.
nitrofurantoin.ti,ab.
penicillin.ti,ab.
tetracycline.ti,ab.
trimethoprim.ti,ab.
synbiotic agent/
exp probiotic agent/
exp bifidobacterium bifidum/
exp Lactobacillus/
exp Bacillus/
exp enterococcus/
exp Escherichia/
exp Saccharomyces/
streptococcus thermophilus/
Bifidobacterium/
synbiotic*.ti,ab.
probiotic*.ti,ab.
bifido*.ti,ab.
lactobacill*.ti,ab.
enterococcus.ti,ab.
escherichia.ti,ab.
saccharomyces.ti,ab.
streptococcus thermophilus.ti,ab.
familact.ti,ab.
probinul neutro.ti,ab.
rebyota.ti,ab.
Vowst.ti,ab.
VE303.ti,ab.
ACHIM.ti,ab.
192 or 193 or 194 or 195 or 196 or 197 or 198 or 199 or 200 or 201 or 202 or 203 or 204 or 205 or 206 or 207 or 208 or 209 or 210 or 211 or 212 or 213 or 214 or 215 or 216 or 217 or 218 or 219 or 220 or 221 or 222 or 223 or 224 or 225 or 226 or 227 or 228 or 229 or 230 or 231 or 232 or 233 or 234 or 235 or 236 or 237 or 238 or 239 or 240 or 241 or 242 or 243 or 244 or 245 or 246 or 247 or 248 or 249 or 250 or 251 or 252 or 253 or 254 or 255 or 256 or 257 or 258 or 259 or 260 or 261 or 262 or 263 or 264 or 265 or 266 or 267 or 268 or 269 or 270 or 271 or 272 or 273
(random$ or placebo$ or single blind$ or double blind$ or triple blind$).ti,ab.
RETRACTED ARTICLE/
or/275-276

(animal$ not human$).sh,hw.
(book or conference paper or editorial or letter or review).pt. not exp randomized controlled trial/
(random sampl$ or random digit$ or random effect$ or random survey or random regression).ti,ab. not exp randomized controlled trial/
191 and 274 and 285
