# Supplementary material for: Efficacy and safety of microbiota-targeted therapeutics in autoimmune and inflammatory rheumatic diseases: protocol for a systematic review and meta-analysis of randomised controlled trials
Source: BMJ Open. 2025 Dec 14;15(12):e101593. doi: 10.1136/bmjopen-2025-101593 (PMC12706220; doi:10.1136/bmjopen-2025-101593)
Supplement: Supplementary file 2 [file bmjopen-2025-101593.reviewer_comments.pdf]

PEER REVIEW HISTORY

BMJ Open publishes all reviews undertaken for accepted manuscripts. Reviewers are asked to complete a checklist review form and are provided with free text boxes to elaborate on their assessment. These free text comments are reproduced below.

ARTICLE DETAILS

Title (Provisional)

Efficacy and safety of microbiota-targeted therapeutics in autoimmune and inflammatory rheumatic diseases: protocol for a systematic review and meta-analysis of randomised controlled trials

Authors

Kragsnaes, Maja Skov; Gilbert, Benoit Thomas P.; Sofíudóttir, Bjørk Khaliqi; Rooney, Christopher Michael; Hansen, Sebrina Maj-Britt; Mauro, Daniele; Mullish, Benjamin H; Bergot, Anne-Sophie; Mankia, Kulveer Singh; Goel, Niti; Bakland, Gunnstein; Johnsen, Peter Holger; Miguens Blanco, Jesus; Li, Simone; Dumas, Emilie; Lage-Hansen, Philip Rask; Wagenaar, Carlijn; Bakdash, Ghaith; Robinson, Mat; Kristiansen, Karsten; Marchesi, Julian R.; Schett, Georg; Zaiss, Mario M.; Orlu, Mine; van Schaadenburg, Dirkjan; Scher, Jose U.; McGonagle, Dennis; Elewaut, Dirk; Breban, Maxime; Tugwell, Peter; Finckh, Axel; Ciccia, Francesco; Kriegel, Martin A.; Daien, Claire; EULAR Study Group MICMUC , ,; Ellingsen, Torkell; Christensen, Robin

---

VERSION 1 - REVIEW

Reviewer 1

Name Essouma, Mickael

Affiliation Faculty of Medicine and Biomedical Sciences, University of Yaoundé I, Yaoundé, Cameroon, Department of Internal Medicine and Specialties

Date 16-Apr-2025

COI None

Myc omments are appended in the attached manuscript.

Available online on 16/04/2025

****** The reviewer provided a marked copy with additional comments. Please contact the publisher for full details. ******

VERSION 1 - AUTHOR RESPONSE

TITLE SECTION

1. Reviewer's comment about the title and terminology.

Response: We thank the reviewer for the suggestion and have incorporated the recommended changes to the title (p. 1). However, we have retained the terminology *"microbiota-targeted interventions"* as it more accurately reflects the scope of therapies examined in this review. This term encompasses a broader range of strategies directed at modulating the microbiota, whereas *"therapies targeting microbiomal dysbiosis"* implies a narrower focus on correcting imbalance alone.

ABSTRACT SECTION

1. Reviewer's suggestion regarding the terminology "microbiome" and "dysbiosis".

Response: We thank the reviewer for the helpful suggestion. We have made the recommended changes (p. 4); however, we have retained the term *"microbiota"* rather than *"microbiome"*, as it more precisely refers to the community of microorganisms themselves rather than their collective genomes(see Marchesi JR, Ravel J. The vocabulary of microbiome research: a proposal. Microbiome. 2015 Jul 30;3:31. doi: 10.1186/s40168-015-0094-5. PMID: 26229597; PMCID: PMC4520061. https://pubmed.ncbi.nlm.nih.gov/26229597/). We have also avoided the term *"dysbiosis"* because it lacks a consistent definition, is scientifically incorrect, and may imply causality that remains uncertain in the context of autoimmune and inflammatory rheumatic diseases (AIRDs).

STRENGTHS AND LIMITATIONS SECTION

1. Reviewer's suggestion regarding the Strengths and Limitations section and GRADE assessments.

Response: We thank the reviewer for their suggestion and have made the recommended grammatical corrections. However, we believe it would be premature to speculate on the expected level of evidence using GRADE assessments, as these can only be conducted once the systematic review is completed. We have, however, highlighted the planned use of GRADE as a methodological strength of the study (p. 5).

INTRODUCTION SECTION

1. Reviewer's suggestion regarding supporting references for the section.

Response: We thank the reviewer for their suggestions. We have revised the section to improve clarity and have added the following references to support our statements (p. 6):

Sarzi-Puttini P, Ceribelli A, Marotto D, Batticciotto A, Atzeni F. Systemic rheumatic diseases: From biological agents to small molecules. Autoimmun Rev. 2019 Jun;18(6):583-592. doi: 10.1016/j.autrev.2018.12.009.

Wang S, Ning W, Tang H, Mu C, Huang X. Efficacy and safety study of targeted small-molecule drugs in the treatment of systemic lupus erythematosus. Arthritis Res Ther. 2024 May 10;26(1):98. doi: 10.1186/s13075-024-03331-8. PMID: 38730460.

Buch MH, Eyre S, McGonagle D. Persistent inflammatory and non-inflammatory mechanisms in refractory rheumatoid arthritis. Nat Rev Rheumatol. 2021 Jan;17(1):17-33. doi: 10.1038/s41584-020-00541-7.

Poddubnyy D, Navarro-Compán V, Torgutalp M, Arends S, Aydin SZ, Battista S, van den Bosch F, Bundy C, Cauli A, Davies J, Dougados M, Duruöz T, El-Zorkany B, Fong W, van Gaalen F, Garcia-Salinas R, Garrido Cumbrera M, Géher P, Gensler L, Grazio S, Huang F, Kishimoto M, Landewé R, Leung YY, Machado PM, Marzo-Ortega H, Meghnathi B, Molto A, Nikiphorou E, Ramiro S, Rudwaleit M, Saad CGS, Sepriano A, Wei J, Baraliakos X, van der Heijde D; ASAS. The Assessment of SpondyloArthritis International Society (ASAS) Consensus-Based Expert Definition of Difficult-to-Manage, including Treatment-Refractory, Axial Spondyloarthritis. Ann Rheum Dis. 2025 Apr;84(4):538-546. doi: 10.1016/j.ard.2025.01.035.

2. Reviewer's comment: Can you revise this statement to elaborate more deeply (go beyond the *Firmicutes* phylum) on the microbiotic microorganisms that have been associated with major autoimmune and other immune-mediated diseases by body site (oral, gut, respiratory, skin, vagina) using a table?

Response: We thank the reviewer for this insightful suggestion. We agree that a comprehensive overview of microbiota alterations across multiple body sites in autoimmune and immune-mediated diseases would provide valuable context. However, such a table would require a detailed synthesis of heterogeneous literature encompassing distinct microbial niches (e.g., oral, gut, skin, respiratory, and vaginal microbiota) and diverse methodological approaches to microbiota profiling. This would extend beyond the scope of the current protocol, which specifically focuses on therapies modulating the gut microbiota and their effects on autoimmune and inflammatory rheumatic diseases. To clarify this scope, we have revised the relevant section of the Introduction to explicitly state that the review will be limited to gut microbiota–targeted interventions (p. 8).

3. Reviewer's comment: Before this statement, are all the microbiome treatment modalities enumerated used for autoimmune and other immune-mediated diseases? If not, why are some used and while the others are not? Along this line, can you draw a table outlining the mechanisms of action of the different microbiome treatment modalities and their uses across the main immune-mediated diseases? What about microbiome mimetics among the microbiome treatment modalities?

Response: We thank the reviewer for this insightful comment. While a comprehensive table summarising the mechanisms of action and applications of all microbiome-targeted interventions across immune-mediated diseases would be informative, this is beyond the scope of the current review, which focuses specifically on therapies targeting the gut microbiota in autoimmune and inflammatory rheumatic diseases (AIRDs).

We have clarified in the text that not all microbiota-targeted interventions are currently applied in AIRDs (p. 8). Interventions such as probiotics, prebiotics, synbiotics, and FMT have been explored clinically in AIRDs, whereas bacteriophages and microbiome mimetics remain emerging modalities under early investigation. The revised paragraph (p. 8) also outlines the potential mechanisms of action of these therapies, including

direct and indirect immunomodulatory effects and modulation of pharmacomicrobiomics. This approach acknowledges the diversity of interventions while keeping the focus on those relevant to our review.

RATIONALE SECTION

Reviewer's comment: In brief, consider revising this statement implementing a table and a figure in the manuscript as requested

Response: We thank the reviewer for this suggestion. While we agree that tabular presentation can sometimes enhance clarity, we believe that, in this instance, the narrative paragraph effectively conveys the importance of assessing methodological quality and the rationale for explicitly stating this objective in the protocol. Retaining the paragraph allows us to emphasize our commitment to transparency and rigor in evaluating risk of bias, which may be less clear in a table format. Therefore, we respectfully propose to keep this section in its current narrative form (pp. 7-8).

METHODS SECTION

OBJECTIVE

1. **Reviewer's comment: If the overarching goal of this systematic review is inform guidelines on the treatment of AIRDs with microbiome-targeted therapies (as it sems in lines 55-60 on page 6), this should be reported under this sub-title as well.**

Are you planning to assess the efficacy of those treatments on AIRD-specific outcomes? Given that those treatments have efficacy endpoints that go beyond immune responses, specify.

1. **Response:** We have implemented the suggested changes (p. 8), including omitting the following two references:

Moher D, Shamseer L, Clarke M, et al. Preferred reporting items for systematic review and meta-analysis protocols (PRISMA-P) 2015 statement. Systematic reviews 2015;4:1. doi: 10.1186/2046-4053-4-1 [published Online First: 2015/01/03]

Christensen R, Bliddal H, Henriksen M. Enhancing the reporting and transparency of rheumatology research: a guide to reporting guidelines. Arthritis research & therapy 2013;15(1):109. doi: 10.1186/ar4145

2. **Reviewer comment**: **Did you wish to say "placebo"?**

Response: We appreciate the reviewer's comment. We intentionally used the broader term *"inactive comparator"* rather than *"placebo"* to encompass all types of control interventions that do not exert a specific therapeutic effect. This includes placebo controls as well as other forms of non-active comparators (e.g., sham or inert interventions), thereby maintaining precision and inclusivity in our study eligibility criteria.

3. **Reviewer comment: Consider removing this statement here because the assessment of the methodological quality of included randomized controlled trials is an integral part of a meta-analysis of randomized controlled trials.**

Response: We thank the reviewer for this comment. We agree that the assessment of methodological quality is a standard and essential component of meta-analyses of randomised controlled trials. However, we believe it is important to explicitly state this objective in the protocol to ensure transparency and clarity regarding the planned methods. Including this statement highlights our commitment to rigorously evaluating the risk of bias and strengthens the methodological framework of the review. Therefore, we respectfully propose to retain the statement in the protocol text (p. 8).

STUDY CRITERIA

1. **Reviewer comment**: **Although RCTs will not be excluded based on their methodological quality, the authors need to consider the sample size as a participant criterion for study inclusion, to ensure they are not going a study with high potential for type 1 or type 2 errors.**

Response: We thank the reviewer for this thoughtful comment. We agree that small sample sizes may increase the risk of type I or type II errors in individual RCTs. However, in line with standard systematic review methodology and PRISMA-P recommendations, we will not exclude trials based on sample size alone to avoid introducing selection bias. Instead, we will assess the impact of study size and precision through the Cochrane Risk of Bias 2 tool and address potential imprecision in the GRADE assessment. Where relevant, sensitivity analyses will be performed to explore the influence of small-sample studies on pooled estimates.

2. **Reviewer's comment: I suggest editing this statement in these likes:**

"**The interventions considered in this systematic review and meta-analysis are those that target the gut microbiome implicated in the development of AIRDs through human and/or animal studies, without restrictions imposed on the dosage, frequency and route of administration, and the duration of treatment:"**

Response: We thank the reviewer for this suggestion. We have considered the proposed wording; however, we prefer to retain our original phrasing as it more accurately reflects the scope and intent of this review. Specifically, our definition emphasises that the therapies target the intestinal microbiota and/or gut barrier function through interventions administered to the gastrointestinal tract with the intention to treat AIRDs via modification of the intestinal *milieu*. We believe this phrasing clearly distinguishes these interventions from broader experimental approaches in human or animal studies, while still maintaining transparency regarding dosage, frequency, duration, and route of administration (pp. 9-10).

3. **Reviewer's comment: Consider revising this statement so that the comparator here is consistent with what is written in the introduction.**

Response: We thank the reviewer for this suggestion. To improve clarity and ensure consistency with the Objective section, we have rephrased this sentence as follows: *"All standard comparator types for the control*

groups, including inactive controls and established disease-modifying anti-rheumatic drugs (DMARDs), will be included." (p. 10)

4. Reviewer's comment: Consider providing these mechanisms of action (for each of these microbiome treatment modalities) with the target microbiotic organisms, frequencies and routes of administration, durations of treatments, and dosages in the proposed table on microbiome treatments for AIRDs.

Response: We thank the reviewer for this suggestion. While we have revised the Introduction to clarify which therapies are relevant to the scope of this review, we prefer to remain explicit about the selected microbiota-targeted interventions and retain their definitions (pp. 9-10). We believe that presenting the interventions in the text, rather than in a table, provides greater clarity and ensures the nuances of each selected therapy are accurately conveyed.

OUTCOMES

1a. Reviewer's comment: Could you revise this statement to just state you will assess primary and secondary outcomes? "For the quantitative evidence synthesis, we will use a hierarchy of pre-set criteria for identifying preferred outcomes to ensure that the most primary endpoint is selected from each study with the least likelihood of selective outcome reporting bias.[68 69]"

1b. Reviewer's comment: What is the hierarchy proposed by Linde et al? This statement is reminiscent of a scoping review meanwhile this is the protocol of a systematic review and meta-analysis of RCTs. You are expected to define the primary outcome/endpoint and the secondary outcomes/endpoints. Because it seems that you engaged in this research process to assess whether microbiome-based therapies are efficaious/effective for AIRDs, the primary endpoint would most likely be the "efficacy of microbiome- based therapies on AIRD-specific endpoints based on clinician and/or patient-reported disease activity measures". This means evaluating the efficacy of microbiome-based therapies on DAS-28 for RA for example, on SLEDAI-2K/other SLE disease activity measure for SLE, on ESSDAI for SS for example, on myositis activity measures for myositis for example, and so on. Secondary endpoints would be treatment safety and main AIRD patient-reported measures reflecting their lived experiences of the disease and how AIRDs affect their quality of life. While providing information about the primary outcome and the secondary outcomes, the authors should provide the time points for outcome evaluation after treatment administration.

Author comments to 1a and 1b: We thank the reviewer for this constructive comment and agree that the distinction between primary and secondary outcomes should be presented more clearly.

In our systematic review and meta-analysis, the primary outcome will be the *trial-specific primary endpoint* as defined in each individual randomised controlled trial. This approach ensures that the analysis respects the methodological design and statistical integrity of each included study, while minimising bias due to selective outcome reporting. We have clarified this explicitly in the revised manuscript (please see the "Primary outcome" section; pp. 10-11).

In cases where the primary outcome is not clearly specified in the original report, we will apply the hierarchy proposed by Linde et al. (Lancet 1997;350(9081):834-43) to identify the most relevant endpoint:

(1) the predefined main outcome used for sample size calculation;
(2) the patient's global assessment of improvement;
(3) the physician's global assessment of improvement; and
(4) an outcome measure considered most clinically relevant by reviewer consensus.

We have also expanded the description of secondary outcomes, specifying the core domains and representative measurement instruments (based on OMERACT recommendations) to ensure consistency across AIRDs. The revised section now clearly distinguishes between primary efficacy outcomes, secondary efficacy outcomes, and safety outcomes, following *BMJ Open* format (pp. 11-13).

Finally, to enhance clarity, we have removed redundant explanatory sentences and refined the structure so that it now clearly outlines the hierarchy and rationale for outcome selection, definitions and examples of primary and secondary outcome domains, and safety outcomes included in the review.

INFORMATION SOURCES

1. **Reviewer's comment: Consider removing subheadings in the "Bibliographic databases" and "Other sources" section.**

Response: We thank the reviewer for this suggestion. We have removed the subheadings and revised the section to improve flow while maintaining clarity and completeness of the search strategy (p. 14).

2. **Reviewer's comment: Are backward and forward citation searching the ultimate tests to assess the sensitivity of literature search in a systematic review and meta-analyses of RCTs?**

Response: We thank the reviewer for this insightful comment. We acknowledge that backward and forward citation searching, while valuable, are not a definitive test of search sensitivity on their own. In our protocol, we have designed a comprehensive database search strategy across multiple sources, incorporating synonyms, MeSH terms, and relevant spelling variants, to maximize retrieval of relevant trials. We consider the combination of this broad search strategy with backward and forward citation searching to be sufficiently rigorous for identifying eligible studies for our systematic review and meta-analysis. This approach balances comprehensiveness with feasibility while adhering to established best practices for systematic review methodology.

SEARCH STRATEGY

1. **Reviewer's comment: You need to add details about the keywords used in the search strategies, state that the EMBASE search strategy provided is a preliminary search strategy (because it may still change), and also provide the preliminary search strategies used in the other searched electronic databases. Furthermore, consider revising the presentation of the preliminary search strategy used in EMBASE and other electronic databases as recommended in the relevant PRISMA checklist (https://www.prisma-statement.org/prisma-search). Accordingly, you are also expected to upload that PRISMA checklist as a supplemental material to highlight how you implemented the content of that checklist in this manuscript.**

Response: We appreciate the reviewer's attention to the transparency and reporting quality of the search strategy. In accordance with *BMJ Open*'s guidance for protocol manuscripts, we have provided the complete and detailed search strategy for Embase as supplementary material. This strategy is now explicitly labelled as *preliminary* (p. 14), as it may be refined further prior to the final search execution.

The search strategies for MEDLINE and Cochrane will be adapted directly from the Embase strategy, with corresponding adjustments for database-specific indexing terms (e.g., MeSH instead of Emtree) and syntax. This is now specified in the search strategy section (p. 14). We believe that providing one comprehensive example (Embase) at this stage ensures both clarity and reproducibility while avoiding unnecessary duplication across databases.

We have also confirmed that the PRISMA-P checklist was included as a supplementary file in the initial submission (March 2025), in accordance with journal requirements.

STUDY RECORDS

1a. Reviewer comment: This information belongs in the "Selection process" sub-section below. The authors should first state that there are two groups of authors who will select the total number of studies (retrieved from literature search) independently from each other before describing the study selection process according to the plan proposed below. Also consider elaborating on how each group is expected to conduct the study selection process, including whether these events will require in-person and/or online meetings.

1b. Reviewer comment: Before these statements, consider describing the data that will be collected to help you assess the primary outcomes and secondary outcomes. Where are those data going to be recorded? Is there a preconceived data abstraction sheet? Will data collectors be trained before undertaking that task? Will there be a pre-test before data extraction start properly?

If the studies will be selected for inclusion by two independent groups, will it not be the same situation for the two groups? Especially since there are many authors.

Response to 1a and 1b: We thank the reviewer for this helpful comment. We agree that the previous version of the *Study records* section contained some overlap and redundancies between the "Data management," "Selection process," and "Data collection process" subsections, which may have affected readability.

In the revised manuscript, this section has been thoroughly streamlined for clarity and consistency while retaining all methodological details (p. 14-16). The revised version now clearly outlines:

1. How Covidence will be used for data management, deduplication, and screening;
2. How two independent reviewers (and a third in case of disagreement) will perform the selection process; and
3. How data extraction and risk of bias assessment will be conducted independently by two reviewer groups using a customised REDCap database, with adjudication by a fifth blinded reviewer if needed.

We thank the reviewer for highlighting the importance of training and calibration among data collectors. In response, we have added a description of a pilot testing phase prior to data extraction, where all reviewers will be trained using a small sample of trials to ensure consistency, reliability, and familiarity with the REDCap data collection form. The revised manuscript now reflects this step in the *Data collection process* section (p. 15).

No methodological changes have been made – only rewording and restructuring to improve flow and alignment with *BMJ Open*'s reporting standards.

DATA ITEMS

Reviewer's comment: This text would better be replaced with a pragmatic description of how the RoB2 tool is used to rate a RCT as a low-quality or a high-quality one in a table inserted in this main manuscript. Through tha table (in the footnote for example), the authors could also expalin in what case the risk of bias of a RCT will be considered to be unclear. The authors could use two RCTs on microbiome-based treatments in AIRDs as examples of high-quality and low-quality trials. See Sterne et al. BMJ 2019;366:l4898 | doi: 10.1136/bmj.l4898

Response: We thank the reviewer for the suggestion regarding a more pragmatic presentation of the RoB 2 assessment. We have opted to retain our current description of the process in the text to maintain clarity and conciseness for the protocol manuscript. While we acknowledge that a table with examples could illustrate how trials are rated, we believe this level of detail is more appropriate for the final systematic review rather than the protocol. Our protocol clearly describes how judgments of low, high, or unclear risk of bias will be made and how disagreements will be resolved. We will apply the RoB 2 tool as outlined, and illustrative examples, as suggested, can be provided in the main review manuscript once the included trials have been evaluated.

DATA SYNTHESIS

Reviewers' comment: This crucial sub-section of the methods section should be thoroughly revised to specify how the main statistical analysis of primary and secondary trial endpoints will be conducted, the softwares that will be used for that analysis, the statistical tests that will be used and why they will use those statistical tests specifically, how between-study heterogeneity assessment will be conducted (including the hetrogeneity classification to be used and the factors presumed to affect that heterogeneity), in what conditions and how subgroup, meta-regression, sensitivity and publication bias analyses will be conducted. It is when reporting on the conduct of statistical analyses for primary and secondary trial endpoints that the authors are expected to elaborated on how they plan to deal with missing data and losses to follow-up. The sub-section should end with details about results presentation and definition of the statistical significance of results from the different tests performed. Consider implementing my comments above trial outcomes in the revision of this sub-section.

Response: We thank the reviewer for the detailed suggestions. In the revised manuscript, the Data Synthesis section now clearly describes how primary and secondary endpoints will be analysed (pp. 17-18), including:

calculation of effect estimates for binary and continuous outcomes (ORs and SMDs), software to be used (R and Stata), and rationale for random-effects models with fixed-effect sensitivity analyses. Between-study heterogeneity will be assessed using I^2 and explored via subgroup and meta-regression analyses. We also describe handling of missing data, ROB-ME assessment for bias due to missing evidence, and evaluation of publication bias with funnel plots and asymmetry tests. Results will be presented as pooled effect estimates with 95% CIs.

We believe these revisions provide a transparent and comprehensive description of our planned statistical analyses while maintaining the pre-specified methodology.

STUDY TIMELINE

We have now provided an outline of the expected study timeline (p. 19):

- Development and finalisation of search strategy: by December 2025
- Title and abstract screening: by February 2026
- Full-text review and data extraction: by June 2026
- Statistical analysis and synthesis of results: by October 2026
- Manuscript preparation and dissemination of findings: by December 2026

PATIENT AND PUBLIC INVOLVEMENT

The statement on patient and public involvement has now been moved to the METHOD section of the main manuscript (p. 19).

DISCUSSION

1. **Reviewer's comment: This information [the first paragraph of the Discussion section] belongs in the introduction more specifically where the rationale for conducting the systematic review and meta-analysis on microbiome-based treatments in AIRD patients is presented.**

Response: We thank the reviewer for this suggestion. We agree that the information in the first paragraph of the Discussion better reflects the rationale for conducting this systematic review and meta-analysis. Accordingly, we have moved this content to the Introduction section to improve the logical flow and context of the manuscript (p. 7).

2a. Reviewer's comment: This resembles the aim of a scoping review meanwhile this is the protocol of a systematic review and meta-analysis.

2b. Remember that this is not the protocol of a scoping review. If the systematic review aims to prepare guidelines on the use of treatments targeting the microbiome in AIRD patients, state that clearly in the introduction and here.

Reponse to 2a and 2b: We thank the reviewer for this observation. We acknowledge that some phrasing in the manuscript may have suggested a scoping approach. We have revised the text to clearly reflect that this is a systematic review and meta-analysis, with a predefined protocol, focused on synthesising evidence from randomised controlled trials to evaluate the efficacy and safety of microbiota-targeted therapies in AIRDs. The objectives now emphasize quantitative evidence synthesis, predefined primary and secondary outcomes, and methodological rigour in line with systematic review standards (pp. 20-22).

TABLE 1

Reviewer's comment: i suggest deleting this table because the eligibility criteria would best be clearly enumerated in the text as seen above.

Response: We thank the reviewer for this suggestion. We agree that presenting the eligibility criteria directly in the manuscript provides a clearer and more streamlined presentation. Accordingly, we have omitted the table to improve readability.

TABLE 2

Reviewer's comment: I do not see the relevance of this table. You need to cite these diseases in the eligibility criteria sub-section of the methods section as commented above. I see autoinflammatory diseases in this table, but I saw the word "autoinflammatory" nowhere in the introduction.
There are many more autoinflammatory and autoimmune diseases dealt with in rheumatology than are mentioned in this table. Consider specifying why you will focus on these diseases.

Response: We thank the reviewer for this comment. We find the table relevant as it explicitly outlines which diseases are included in this systematic review, providing clarity for readers. This table is now named Table 1 in the revised version of the manuscript (pp. 24-25).

FIGURE 1

1. **Reviewer's comment: This box suggests that you are planning to perform manual searches in addition to the traditional electronic literature search and the "pragmatic search". How, you did not mention the manual search above when describing the search strategies.**

Response: We thank the reviewer for this observation. The manual search is described under the *Information sources* section: "After inclusion of eligible studies, a backward and forward citation search will be conducted in Scopus for twenty randomly selected reports. If no additional relevant records are identified, the search will be considered comprehensive. If new studies are found, full citation tracking will be conducted for all included reports to capture any remaining relevant records." To ensure consistency, we have now clarified in Figure 1 that this refers specifically to backward and forward citation searching.

2. **Reviewer's comment: Why this exclusion criterion? Are you not going to include any type of article be it the abstract only or the abstract with a full-text?**

Response: We agree with the reviewer's point. Studies available only as abstracts will be considered for inclusion; however, they will be included in the meta-analysis only if sufficient data are available for analysis.

This has now been clarified in the figure legend (p. 26-27).

3. **Reviewer's comment: Consider revising this figure to subdivide it on the left into three parts (as seen with the PRISMA flow chart of study selection): identification, screening and included.**

Response: We thank the reviewer for this suggestion. Figure 1 has been revised accordingly (p. 26), with different box shadings representing the three PRISMA stages: dark grey for *identification*, light blue for *screening*, and white for *inclusion*.

We believe these revisions address the reviewer's concerns and improve the clarity and alignment of Figure 1 with the PRISMA-P framework.

Closing author remark

We sincerely thank the reviewer for thoughtful and constructive feedback, which has helped us improve the clarity, rigor, and transparency of our protocol. We believe that all comments have been addressed in the revised manuscript, and we hope that the changes made now meet the expectations of the editor and reviewers.

VERSION 2 - REVIEW

Reviewer 1

Name Essouma, Mickael

Affiliation Faculty of Medicine and Biomedical Sciences, University of Yaoundé I, Yaoundé, Cameroon, Department of Internal Medicine and Specialties

Date 14-Nov-2025

COI

The authors have improved the manuscript. However, there are still methodological issues that should be dealt with.

Given that it is now clear that the authors focus on gut microbiota-directed therapies, consider making this specification in the manuscript's title.

In my opinion, both the primary and secondary efficacy and safety outcomes should be your choice because you know why you conduct the systematic review. That is why there are eligibility criteria and the indirectness criterion in the GRADE assessment of the strength of evidence of systematic reviews (Journal of Clinical Epidemiology 66 (2013) 151e157).

Sjogren syndrome should read Sjogren disease in table 1 (see https://www.nature.com/articles/s41584-025-01268-z).

Page 12 of 102, lines 15-17 I understand, but it remains somewhat discrepant with usual rheumatology research practice: see https://doi.org/10.1002/acr.22583.

You claimed that a p-value threshold of 0.05 will be used to define the statistical significance of results. However, in meta-analyses, the p-value threshold varies across different analyses. See for example https://www.sciencedirect.com/science/article/pii/S221339841830099X.

I did not perform any language assessment in line with the journal's policy for peer review.

Best wishes

Available online on 14/11/25

Mickael Essouma, M.D.

VERSION 2 - AUTHOR RESPONSE

REVIEWER 1

1: Given that it is now clear that the authors focus on gut microbiota-directed therapies, consider making this specification in the manuscript's title.

Response: We thank the reviewer for the helpful suggestion regarding terminology. While we appreciate the recommendation to use the term *microbiota-directed therapies*, we respectfully propose to retain *microbiota-targeted therapies* in the title and throughout the manuscript.

The interventions included in this review – probiotics, synbiotics, postbiotics, faecal microbiota transplantation (FMT), live biotherapeutic products, and antibiotics – are all intended to *modulate or target* the gut microbiota, either by altering microbial composition, influencing microbial function, or replacing microbial communities. In the literature, these modalities are commonly grouped under the umbrella term *microbiota-targeted therapies*, reflecting their shared therapeutic aim of modifying the microbiota to achieve clinical benefit.

In contrast, *microbiota-directed therapies* is often used in a more specific context, describing interventions that are designed through defined mechanistic pathways to exert precise effects on microbial functions. Because our review encompasses a broader and more heterogeneous category of microbiota-modulating strategies – including modalities that are not mechanistically “directed” – we believe that *microbiota-targeted therapies* accurately reflects the scope and intent of the interventions under investigation.

2. In my opinion, both the primary and secondary efficacy and safety outcomes should be your choice because you know why you conduct the systematic review. That is why there are eligibility criteria and the indirectness criterion in the GRADE assessment of the strength of evidence of systematic reviews (Journal of Clinical Epidemiology 66 (2013) 151e157).

Response: We thank the reviewer for the thoughtful and important comment regarding the need for the review team—not the individual trials—to define the primary and secondary outcomes of interest. We fully agree with the principle raised. This aligns with standard systematic review methodology and with the GRADE guidance on specifying outcomes relevant to the decision context and on evaluating indirectness.

In response, we have substantially revised the Outcomes section (pages 10-11).

Specifically:

1. We now explicitly state that the primary and secondary outcomes are *prespecified by the review team*, based on the purpose of the review and consistent with GRADE principles.
    - Previously, the text could be interpreted as if the trial's stated primary outcome determined the review's primary endpoint.
    - We now clarify that the review-defined primary outcome guides evidence synthesis.
2. We retained a hierarchy only to identify the best corresponding outcome from each trial, not to define the primary outcome itself.
    - The revised text explains that the hierarchy (sample-size outcome → patient global → physician global → reviewer-judged) is used solely to select the most relevant measure within each study when multiple candidate outcomes exist, thereby reducing selective reporting bias.
3. We now explicitly justify why our chosen primary outcome best reflects the review's objective – namely, assessing the efficacy of microbiota-targeted therapies for AIRDs.
4. For secondary outcomes, we now clearly state that these are pre-specified by the review team, drawing on OMERACT core domain frameworks because no universal AIRD core set exists.
    - The previous version emphasized heterogeneity in trial reporting; the revised version emphasizes *reviewer-defined outcome domains* with trials contributing data only where available.
5. We clarified that trial eligibility does not depend on outcome reporting, but participation in specific meta-analyses requires extractable data.
    - This improves transparency and aligns with standard systematic review practice.

Overall, these revisions ensure that the outcomes used in the review are explicitly *ours*, grounded in methodological guidance, and selected consistently across heterogeneous trials. We are grateful to the reviewer for prompting this important clarification.

3. Sjogren syndrome should read Sjogren disease in table 1 (see https://www.nature.com/articles/s41584-025-01268-z).

Response: We thank the reviewer for this helpful correction. We agree with the suggested terminology and have updated Table 1 to use *Sjögren disease* accordingly (Table 1).

4. Page 12 of 102, lines 15-17 I understand, but it remains somewhat discrepant with usual rheumatology research practice: see https://doi.org/10.1002/acr.22583.

Response: We thank the reviewer for this comment. We were not entirely certain which passage the reviewer was referring to on page 12, lines 15–17 (these lines fall within a section break in the revised manuscript). We therefore assume the comment relates to page 9, lines 15–17, which state: *"Eligible studies must include individuals diagnosed with one of the listed AIRDs (Table 1). The term 'diagnosis' will be used rather than clinical classification criteria to match clinical practice in early rheumatology intervention."*

We appreciate the reviewer's point regarding usual rheumatology research practice, where clinical trials commonly enrol well-characterised patient populations defined by established diagnostic or, where necessary, classification criteria in accordance with EULAR/ACR recommendations. Our intention is not to deviate from these standards but rather to avoid excluding earlier randomised trials that were conducted before the most recent classification criteria were published.

To address this potential discrepancy, we have clarified the text to state that we will accept diagnoses based on recognised diagnostic criteria or established classification criteria available at the time the study was conducted. This approach allows us to maintain methodological consistency with rheumatology research practice while ensuring that relevant earlier trials are not inadvertently excluded.

Here is the revised text: "Eligible studies must include individuals diagnosed with one of the AIRDs listed in Table 1. To ensure consistency with rheumatology research practice while avoiding unnecessary exclusion of earlier trials, we will accept diagnoses based on recognised diagnostic criteria or established classification criteria available at the time the study was conducted. If no classification criteria were available for a given condition at the time, we will accept a clinical diagnosis, reflecting routine practice in early rheumatology intervention. No restrictions will be applied on age or sex/gender." (page 9)

We hope this clarification resolves the reviewer's concern.

5. **You claimed that a p-value threshold of 0.05 will be used to define the statistical significance of results. However, in meta-analyses, the p-value threshold varies across different analyses. See for example https://www.sciencedirect.com/science/article/pii/S221339841830099X.**

Response: We thank the reviewer for this important comment. In the revised manuscript,

we have clarified our approach to reporting statistical significance (p. 19). Specifically, we state that exact p-values will be reported for all outcomes, with statistical significance defined as $p<0.05$. We also conduct sensitivity and subgroup analyses alongside the main analyses to facilitate a comprehensive interpretation of the robustness and consistency of findings.

By reporting exact p-values in combination with effect estimates and 95% confidence intervals, we aim to provide sufficient transparency and allow readers to interpret the strength of evidence, in line with current best practices in meta-analysis.
